# Current status and ongoing needs for the teaching and assessment of clinical reasoning – an international mixed-methods study from the students` and teachers` perspective

**DOI:** 10.1186/s12909-024-05518-8

**Published:** 2024-06-05

**Authors:** F. L Wagner, M. Sudacka, A. A Kononowicz, M. Elvén, S. J Durning, I. Hege, S. Huwendiek

**Affiliations:** 1 Institute for Medical Education, Department for Assessment and Evaluation, University of Bern, Bern, Switzerland; 2 Center of Innovative Medical Education, Department of Medical Education, Jagiellonian University, Kraków, Poland; 3 Faculty of Medicine, Department of Bioinformatics and Telemedicine, Jagiellonian University, Kraków, Poland; 4 School of Health, Care and Social Welfare, Mälardalen University, Västerås, Sweden; 5 Faculty of Medicine and Health, School of Health Sciences, Örebro University, Örebro, Sweden; 6 Uniformed Services University of the Health Sciences, Bethesda, MD USA; 7 Institute of Medical Education, University Hospital, LMU Munich, Munich, Germany

**Keywords:** Clinical reasoning, Teaching, Assessment, Needs, Teachers, Students

## Abstract

**Background:**

Clinical reasoning (CR) is a crucial ability that can prevent errors in patient care. Despite its important role, CR is often not taught explicitly and, even when it is taught, typically not all aspects of this ability are addressed in health professions education. Recent research has shown the need for explicit teaching of CR for both students and teachers. To further develop the teaching and learning of CR we need to improve the understanding of students' and teachers' needs regarding content as well as teaching and assessment methods for a student and trainer CR curriculum.

**Methods:**

Parallel mixed-methods design that used web-surveys and semi-structured interviews to gather data from both students (n_survey_ = 100; n_interviews_ = 13) and teachers (n_survey_ = 112; n_interviews_ = 28). The interviews and surveys contained similar questions to allow for triangulation of the results. This study was conducted as part of the EU-funded project DID-ACT (https://did-act.eu).

**Results:**

Both the surveys and interview data emphasized the need for content in a clinical reasoning (CR) curriculum such as “gathering, interpreting and synthesizing patient information”, “generating differential diagnoses”, “developing a diagnostic and a treatment plan” and “collaborative and interprofessional aspects of CR”. There was high agreement that case-based learning and simulations are most useful for teaching CR. Clinical and oral examinations were favored for the assessment of CR. The preferred format for a train-the-trainer (TTT)-course was blended learning. There was also some agreement between the survey and interview participants regarding contents of a TTT-course (e.g. teaching and assessment methods for CR). The interviewees placed special importance on interprofessional aspects also for the TTT-course.

**Conclusions:**

We found some consensus on needed content, teaching and assessment methods for a student and TTT-course in CR. Future research could investigate the effects of CR curricula on desired outcomes, such as patient care.

## Introduction

Clinical reasoning (CR) is a universal ability that mobilizes integration of necessary fundamental knowledge while delivering high-quality patient care in a variety of contexts in a timely and effective way [1, 2]. Daniel et al. [3] define it as a “skill, process or outcome wherein clinicians observe, collect, and interpret data to diagnose and treat patients”. CR encompasses health professionals thinking and acting in patient assessment, diagnostic, and management processes in clinical situations, taking into account the patient ‘s specific circumstances and preferences [4]. How CR is defined can vary between health professions, but there are also similarities [5]. Poor CR is associated with low-quality patient care and increases the risk of medical errors [6]. Berner and Graber [7] suggested that the rate of diagnostic error is around 15%, underlining the threat that insufficient CR ability poses to patient safety as well as increasing healthcare costs [8]. Despite the importance of CR, it appears to be rarely taught or assessed explicitly, often only parts of the CR process are covered in existing curricula, and there seems to be a lack of progression throughout curricula (e.g. [9–14].). Moreover, teachers are often not trained to explicitly teach CR, including explaining their own reasoning to others [10–12] although this appears to be an important factor in the implementation of a CR curriculum [15]. Some teachers even question whether CR can be explicitly taught [16]. Considering these findings, efforts should be made to incorporate explicit teaching of CR into health care professions curricula and training for teachers should be established based on best evidence. However, to date, little is known about what a longitudinal CR curriculum should incorporate to meet the needs of teachers and students.

Insights regarding teaching CR were provided from a global survey by Kononowicz et al. [10], who reported a need for a longitudinal CR curriculum. However, the participants in their study were mainly health professions educators, leaving the needs of students for a CR curriculum largely unknown. As students are future participants of a CR curriculum, their needs should also be investigated. Kononowicz et al. [10] also identified a lack of qualified faculty to teach CR. A train-the-trainer course for CR could help reduce this barrier to teaching CR. To the best of our knowledge, in addition to the work by Kononowicz et al. [10], no research exists yet that addresses the needs of teachers for such a course, and Kononowicz et al. [10] did not investigate their needs beyond course content. Recently, Gupta et al. [12] and Gold et al. [13] conducted needs analyses regarding clinical reasoning instruction from the perspective of course directors at United States medical schools, yet a European perspective is missing. Thus, our research questions were the following:

1. What aspects of clinical reasoning are currently taught and how important are they in a clinical reasoning curriculum according to teachers and students?
2. What methods are currently used to teach and assess clinical reasoning and which methods would be ideal according to teachers and students?
3. In what study year does the teaching of clinical reasoning currently begin and when should it ideally begin according to teachers and students?
4. How should a train-the-trainer course for teachers of clinical reasoning be constructed regarding content and format?

## Methods

### Design

In this study, we used a convergent parallel mixed-methods design [17] within a pragmatic constructivist case study approach [18]. We simultaneously collected data from students and educators using online questionnaires and semi-structured interviews to gain deeper insight into their needs on one particular situation [19]– the development of a clinical reasoning curriculum—to address our research questions. To help ensure that the results of the survey and the interviews could be compared and integrated, we constructed the questions for the survey and the interviews similarly with the exception that in the interviews, the questions were first asked openly. The design was parallel both in that we collected data simultaneously and also constructed the survey and interviews to cover similar topics. We chose this approach to obtain comprehensive answers to the research questions and to facilitate later triangulation [17] of the results.

### Context of this study

We conducted this study within the EU-funded (Erasmus + program) project DID-ACT (“Developing, implementing, and disseminating an adaptive clinical reasoning curriculum for healthcare students and educators”; https://did-act.eu). Institutions from six European countries (Augsburg University, Germany; Jagiellonian University in Kraków, Poland; Maribor University, Slovenia; Örebro University, Sweden; University of Bern, Switzerland; EDU, a higher medical education institution based in Malta, Instruct GmbH, Munich, Germany) with the support of associate partners (e.g., Prof. Steven Durning, Uniformed Services University of the Health Sciences, USA; Mälardalen University, Sweden.) were part of this project. For further information, see https://did-act.eu/team-overview/team/. In this project, we developed an interprofessional longitudinal clinical reasoning curriculum for students in healthcare education and a train-the-trainer course for health profession educators. The current curriculum (for a description of the curriculum, see Hege et al. [20]) was also informed by this study. This study was part of the Erasmus + Knowledge Alliance DID-ACT (612,454-EPP-1–2019-1-DE-EPPKA2-KA).

### Target groups

We identified two relevant target groups for this study, teachers and students, which are potential future users and participants of a train—the—trainer (TTT-) course and a clinical reasoning curriculum, respectively. The teacher group also included individuals who were considered knowledgeable regarding the current status of clinical reasoning teaching and assessment at their institutions (e.g. curriculum managers). These specific participants were individually selected by the DID-ACT project team to help ensure that they had the desired level of expertise. The target groups included different health professions from a large number of countries (see Table 1), as we wanted to gather insights that are not restricted to one profession.

## Development of data collection instruments

### Development of questions

The questions in this study addressed the current status and needs regarding content, teaching, and assessment of clinical reasoning (CR). They were based on the questions used by Kononowicz et al. [10] and were expanded to obtain more detailed information. Specifically, regarding CR content, we added additional aspects (see Table 8 in the Appendix for details). The contents covered in this part of the study also align with the five domains of CR education (clinical reasoning concepts, history and physical examination, choosing and interpreting diagnostic tests, problem identification and management and shared decision-making) that were reported by Cooper et al. [14]. It has been shown that there are similarities between professions regarding the definition of CR (e.g. history taking or an emphasis on clinical skills), while nurses placed greater importance on a patient-centered approach [5]. We aimed to cover as many aspects of CR in the contents as possible to represent these findings. We expanded the questions on CR teaching formats to cover a broader range of formats. Furthermore, two additional assessment methods were added to the respective questions. Finally, one aspect was added to the content questions for a train-the-trainer course (see Table 8 in the Appendix). As a lack of qualified faculty to teach CR was identified in the study by Kononowicz et al. [10], we added additional questions on the specific needs for the design of a CR train-the-trainer course beyond content. Table 8 in the Appendix shows the adaptations that we made in detail.

We discussed the questions within the interprofessional DID-ACT project team and adapted them in several iterative cycles until the final versions of the survey questionnaire and the interview guide were obtained and agreed upon. We tested the pre-final versions with think-alouds [21] to ensure that the questions were understandable and interpreted as intended, which led to a few changes. The survey questionnaires and interview-guides can be found at https://did-act.eu/results/ and accessed via links in table sections D1.1a (survey questions) and D1.1b (interview guides), respectively. Of these questions, we included only those relevant to the research questions addressed in this study. The questions included in this study can be found in the Appendix in Table8.

### Survey

Teachers were asked questions about all content areas, but only the expert subgroup was asked to answer questions on the current situation regarding the teaching and assessment of clinical reasoning at their institutions, as they were considered the best informed group on the matter. Furthermore, students were not asked questions on the train-the-trainer course. Using the abovementioned procedures, we also hoped to improve the response rate as longer surveys were found to be associated with lower response rates [22].

### Interviews

We created two different versions of the interview guide, one for teachers and one for students. The student interview guide did not contain questions on the current status of clinical reasoning teaching and assessment or questions about the train-the-trainer course. The interview guides were prepared with detailed instructions to ensure that the interviews were conducted in a comparable manner at all locations. By using interviews, we intended to obtain a broad picture of existing needs. Individual interviews further allowed participants to speak their own languages and thus to express themselves naturally and as precisely as possible.

### Reflexivity statement

Seven researchers representing different perspectives and professions form the study team. MS has been a PhD candidate representing the junior researcher perspective, while also experienced researchers with a broad background in clinical reasoning and qualitative as well as quantitative research are part of the team (SD, SH, AK, IH, ME, FW). ME represents the physiotherapist perspective, SD, SH, and MS represent the medical perspective. We discussed all steps of the study in the team and made joint decisions.

## Data collection and analysis

### Survey

The survey was created using LimeSurvey software (LimeSurvey GmbH). The survey links were distributed via e-mail (individual invitations, posts to institutional mailing lists, newsletters) by the DID-ACT project team and associate partners (the target groups received specific links to the online-survey). The e-mail contained information on the project and its goals. By individually contacting persons in the local language, we hoped to increase the likelihood of participation. The survey was anonymous. The data were collected from March to July 2020.

### Interviews

Potential interview participants were contacted personally by the DID-ACT project team members in their respective countries. We used a convenience sampling approach by personally contacting potential interview partners in the local language to motivate as many participants as possible. With this approach we also hoped to increase the likelihood of participation. The interviews were conducted in the local languages also to avoid language barriers and were audio-recorded to help with the analysis and for documentation purposes. Most interviews were conducted using online meeting services (e.g. Skype or Zoom) because of restrictions due to the ongoing coronavirus pandemic that occurred with the start of data collection at the beginning of the DID-ACT project. The data were collected from March to July 2020. All interview partners provided informed consent.

### Ethics approval and consent to participate

We asked the Bern Ethics Committee to approve this multi-institutional study. This type of study was regarded as exempt from formal ethical approval according to the regulations of the Bern Ethics Committee (‘Kantonale Ethikkommission Bern’, decision Req-2020–00074). All participants voluntarily participated and provided informed consent before taking part in this study.

### Data analysis

Descriptive analyses were performed using SPSS statistics software (version 28, 2021). Independent samples t-tests were computed for comparisons between teachers and students. When the variances of the two groups were unequal, Welch’s test was used. Bonferroni correction of significance levels was used to counteract alpha error accumulation in repeated tests. The answers to the free text questions were screened for recurring themes. There were very few free-text comments, typically repeating aspects from the closed questions, hence, no meaningful analysis was possible. For this reason, the survey comments are mentioned only where they made a unique contribution to the results.

The interviews were translated into English by the partners. An overarching summarizing qualitative content analysis [23] of the data was conducted. A summarizing content analysis is particularly useful when the content level of the material is of interest. Its goal is to reduce the material to manageable short texts in a way that retains the essential meaning [23]. The analysis was conducted first by two of the authors of the study (FW, SH) and then discussed by the entire author team. The analysis was carried out as an iterative process until a complete consensus was reached within the author team.

The results from the surveys and interviews were compared and are presented together in the results section. The qualitative data are reported in accordance with the standards for reporting qualitative research (SRQR, O’Brien et al. [24]).

## Results

### Sample

Table 1 shows the professional background and country of the interviewees and survey samples. The survey was opened by 857 persons, 212 (25%) of whom answered the questions included in this study. The expert sub-group of teachers who answered the questions on the current status of clinical reasoning teaching and assessment encompassed 45 individuals.

**Table 1:** Interview and survey samples. All values are percentages

	**Interviews**		**Survey**	
	Teachers (*n* = 28)	Students (*n* = 13)	Teachers (*n* = 112)	Students (*n* = 100)
**Background**				
Medicine	54	92	83	88
Nursing	29	8	7	3
Physiotherapy	7	–	2	6
Occupational Therapy	3	–	2	1
Veterinary Medicine	7	–	–	–
Other		–	6	2
**Country**				
Algeria	–	–	1	–
Austria	–	–	1	–
Canada	3	–	2	–
Czechia	–	–	1	–
China (Hong Kong)	–	–	–	2
Germany	36	62	17	27
Malta	–	–	1	5
Netherlands	–	–	3	–
Poland	11	8	15	1
Slovakia	–	–	1	–
Slovenia	7	22	19	31
Solomon Islands	–	–	1	–
Sweden	32	–	12	7
Switzerland	11	8	3	27
Tanzania	–	–	1	–
United States	–	–	22	–
**Year of study**	–		–	
Year 1	–	–	–	16
Year 2	–	–	–	8
Year 3	–	23	–	14
Year 4	–	15	–	9
Year 5	–	23	–	22
Year 6	–	38	–	22
Year 7	–	–	–	2

### Content of a clinical reasoning curriculum for students

The survey results show that “Gathering, interpreting, and synthesizing patient information”, is currently most extensively taught, while “Theories of clinical reasoning” are rarely taught (see Table 2). In accordance with these findings, “Gathering, interpreting, and synthesizing patient information” received the highest mean importance rating for a clinical reasoning curriculum while “Theories of clinical reasoning” received the lowest importance rating. Full results can be found in Table 9 in the Appendix.

**Table 2:** Survey: Importance of current clinical reasoning contents for a longitudinal clinical reasoning curriculum

**Questions: In your curriculum, which of the following aspects are taught? / Please rate the importance of inclusion of each of the following aspects in the envisioned longitudinal curriculum on clinical reasoning**	**Currently taught to a great extent**^a^** (%)**	**Mean importance rating for a CR curriculum**^b^** by Teachers**	**Mean importance rating for a CR curriculum**^b^** by Students**	**Mean importance rating for a CR curriculum**^b^** Total**	P^***^
1. Gathering, interpreting, and synthesizing patient information^****^	59	6.84	6.48	6.67	.000
2. Generating differential diagnoses including defining and discriminating features	53	6.67	6.38	6.53	.012
3. Developing a treatment/management plan	55	6.55	6.30	6.43	.032
4. Developing a diagnostic plan	55	6.53	6.30	6.42	.049
5. Self-reflection on clinical reasoning performance and strategies for future improvement	25	6.50	6.09	6.30	.005
6. Errors in the clinical reasoning process and strategies to avoid them	17	6.37	6.09	6.23	.048
7. Developing a problem formulation/hypothesis^****^	33	6.41	5.93	6.18	.000
8. Interprofessional aspects of clinical reasoning	14	6.18	5.83	6.01	.019
9. Aspects of patient participation in clinical reasoning (e.g. shared decision making)	32	6.17	5.81	6.00	.019
10. Collaborative aspects of clinical reasoning	14	6.15	5.81	5.99	.016
11. Strategies to learn clinical reasoning (e.g. heuristics, rule out worst case scenario)	20	6.01	5.75	5.88	.092
12. Theories of clinical reasoning (e.g. knowledge encapsulation, illness scripts, narrative reasoning)	2	5.68	5.21	5.45	.007

^***^p-values of the comparisons of the importance ratings between teachers and students

^****^Significant difference in importance ratings between teachers and students (*p* < .001). The alpha level for significance was adjusted for multiple testing according to Bonferroni, resulting in a significance level of .0042

^a^Full scale: To a great extent; To some extent; A little; Not at all

^b^Scale: 7 = Very important; 6 = Important; 5 = Somewhat important; 4 = Neutral; 3 = Rather unimportant; 2 = Unimportant; 1 = Very unimportant

Teachers and students differed significantly in their importance ratings of two content areas, “Gathering, interpreting, and synthesizing patient information” (*t*(148.32) = 4.294, *p* < 0.001,* d* = 0.609) and “Developing a problem formulation/hypothesis” (*t*(202) = 4.006, *p* < 0.001,* d* = 0.561), with teachers assigning greater importance to both of these content areas.

The results from the interviews are in line with those from the survey. Details can be found in Table 12 in the Appendix.

### Clinical reasoning teaching methods

The survey participants reported that, most often, case-based learning is currently applied in the teaching of clinical reasoning (CR). This format was also rated as most important for teaching CR (see Table 3). Full results can be found in Table 10 in the Appendix.

Teachers and students differed significantly in their importance ratings of Team-based learning (*t*(202) = 3.079, *p* = 0.002, *d* = 0.431), with teachers assigning greater importance to this teaching format.

Overall, the interviewees provided very similar judgements to the survey participants. Next to the teaching formats shown in Table 3, some of them would employ blended learning, and clinical teaching formats such as bedside teaching and internships were also mentioned. Details can be found in the Appendix in Table 13. In addition to the importance of each individual teaching format, it was also argued that all of the formats can be useful because they all are meant to reach different objectives and that there is not one single best format for teaching CR.

**Table 3:** Survey: Importance of current clinical reasoning teaching formats for a longitudinal clinical reasoning curriculum

**Questions: How is clinical reasoning taught in your curriculum in sessions with a main focus on clinical reasoning? / Please rate the importance of inclusion of each of the following formats in the envisioned** **longitudinal curriculum on clinical reasoning**	**Currently used to a great extent**^a^** (%)**	**Mean importance rating for a CR curriculum**^b^** by Teachers**	**Mean importance rating for a CR curriculum**^b^** by Students**	**Mean importance rating for a CR curriculum**^b^** Total**	P^***^
1. Case-based Learning	44	6.55	6.41	6.48	.225
2. Human simulated patients	26	6.07	6.09	6.08	.891
3. Team-based Learning^****^	17	6.19	5.72	5.96	.002
4. Problem-based Learning (PBL)	26	5.97	5.92	5.95	.758
5. High-fidelity simulation (mannequins)	7	5.77	5.68	5.73	.618
6. Virtual Patients (interactive online cases)	7	5.75	5.41	5.59	.057
7. Lectures	16	4.93	5.35	5.14	.034

^***^p-values of the comparisons of the importance ratings between teachers and students

^****^Significant difference in importance ratings between teachers and students (*p* < .001). The alpha level for significance was adjusted for multiple testing according to Bonferroni, resulting in a significance level of .007

^a^Full scale: To a great extent; To some extent; A little; Not at all

^b^Scale: 7 = Very important; 6 = Important; 5 = Somewhat important; 4 = Neutral; 3 = Rather unimportant; 2 = Unimportant; 1 = Very unimportant

### Start of clinical reasoning teaching in curricula

Most teachers (52.5%) reported that currently, the teaching of clinical reasoning (CR) starts in the first year of study. Most often (46.4%) the participants also chose the first study year as the optimal year for starting the teaching CR. In accordance with the survey results, the interviewees also advocated for an early start of the teaching of CR. Some interview participants who advocated for a later start of CR teaching suggested that the students first need a solid knowledge base and that once the clinical/practical education starts, explicit teaching of CR should begin.

### Assessment of clinical reasoning

The survey results suggest that currently written tests or clinical examinations are most often used, while Virtual Patients are used least often (see Table 4). Despite written tests being the most common current assessment format, they received the lowest importance rating for a future longitudinal CR curriculum. Full results can be found in Table 11 in the Appendix.

**Table 4:** Survey: Importance of current clinical reasoning assessment formats for a longitudinal clinical reasoning curriculum

**Questions: How is clinical reasoning assessed in your curriculum? / Which of these assessment formats should be implemented in the envisioned longitudinal curriculum on clinical reasoning?**	**Currently used to a great extent**^a^** (%)**	**Mean importance rating for a CR curriculum**^b^** by Teachers**	**Mean importance rating for a CR curriculum**^b^** by Students**	**Mean importance rating for a CR curriculum**^b^** Total**	P^***^
1. Clinical examinations (e.g. Objective Structured Clinical Examination or other practical examinations)^****^	38	6.52	6.19	6.36	.005
2. Workplace-based assessments (e.g. Mini-CEX, summative approach)^****^	18	6.27	5.88	6.08	.009
3. Oral examination	20	5.80	5.71	5.75	.611
4. Assessment using virtual patients	15	5.82	5.60	5.71	.203
5. Written test (e.g. multiple choice questions, key feature approach, script concordance tests)	48	5.30	5.03	5.17	.175

^***^p-values of the comparisons of the importance ratings between teachers and students

^****^Significant difference in importance ratings between teachers and students (*p* < .001). The alpha level for significance was adjusted for multiple testing according to Bonferroni, resulting in a significance level of .01

^a^Full scale: To a great extent; To some extent; A little; Not at all

^b^Scale: 7 = Very important; 6 = Important; 5 = Somewhat important; 4 = Neutral; 3 = Rather unimportant; 2 = Unimportant; 1 = Very unimportant

Teachers and students differed significantly in their importance ratings of clinical examinations (*t*(161.81) = 2.854, *p* = 0.005, *d* = 0.413) and workplace-based assessments (*t*(185) = 2.640, p = 0.009, *d* = 0.386) with teachers assigning greater importance to both of these assessment formats.

The interviewees also placed importance on all assessment methods but found it difficult to assess CR with written assessment methods. The students seemed to associate clinical examinations more with practical skills than with CR. Details can be found in the Appendix in Table 14. Two of the interview participants mentioned that CR is currently not assessed at their institutions, and one person mentioned that students are asked to self-reflect on their interactions with patients and on potential improvements.

### Train-the-trainer course

The following sections highlight the results from the needs analysis regarding a train-the-trainer (TTT-) course. The questions presented here were posed only to the teachers.

Most survey participants reported that there is currently no TTT- course on clinical reasoning at their institution but that they think such a course is necessary (see Table 5). The same was also true for the interviewees (no TTT- course on clinical reasoning existing but need for one).

**Table 5:** Survey: Existence and perceived necessity of a clinical reasoning train-the-trainer course

	**Do you have a train-the-trainer course on clinical reasoning at your institution? (%)**	**Do you think the DID-ACT train-the-trainer course is necessary for healthcare educators at your institution? (%)**
Yes	18	78
No	68	4
Don’t know	14	18

In the interviews, 22 participants (78.6%) answered that a TTT-course is necessary for healthcare educators, two participants answered that no such course was necessary, and two other participants were undecided about its necessity. At none of the institutions represented by the interviewees, a TTT-course for teaching clinical reasoning exists.

When asked what the best format for a clinical reasoning TTT- course would be (single answer question), the majority of the survey participants favored a blended learning / flipped classroom approach, a combination of e-learning and face-to-face meetings. (see Table 6).

**Table 6:** Best format for a train-the-trainer course informed by interviews and survey

Format	Summary of interview comments	% Survey
Blended learning/flipped classroom approach (combination of e-learning and face-to-face meetings)	The interviewees think that blended learning would work well. Also, learning together in an interprofessional setting could be beneficial for the teachers and it could allow for them to experience the methods they may later use themselves	57
Series of face-to-face meetings	The interviewees argued that this would need to be reasonably planned regarding the time that teachers can invest	29
E-learning course	There may not be enough motivation from the teachers for this to work well	9
One time face-to-face meeting	The interviewees argued that a single meeting would not be enough for this purpose	3

In the survey comments it was noted that blended-learning encompasses the benefits of both self-directed learning and discussion/learning from others. It would further allow teachers to gather knowledge about CR first in an online learning phase where they can take the time they need before coming to a face-to-face meeting.

The interviewees also found a blended-learning approach particularly suitable for a TTT-course. An e-learning course only was seen as more critical because teachers may lack motivation to participate in an online-only setting, while a one-time face-to-face meeting would not provide enough time. In some interviews, it was emphasized that teachers should experience themselves what they are supposed to teach to the students and also that the trainers for the teachers need to have solid education and knowledge on clinical reasoning.

Table 7 shows the importance ratings of potential content of a TTT-course generated from the survey. To elaborate on this content, comments by the interviewees were added. On average, all content was seen as (somewhat) important with teaching methods on the ward and/or clinic receiving the highest ratings. Some interviewees also mentioned the importance of interprofessional aspects and interdisciplinary understanding of CR. In the survey comments, some participants further expressed their interest in such a course.

**Table 7:** Importance ratings of possible TTT-course content from the survey and comments from the interviews

**Content**	**Summary of interview comments**	**Mean importance rating for a future CR train-the-trainer course**^a^
1. Teaching methods on the wards and/or clinic	Teachers should have a large toolkit to select the most suitable methods from	6.55
2. Clinical reasoning strategies	Experienced teachers are often not aware of their reasoning, so this is also important for teachers	6.48
3. Common errors in the clinical reasoning process	No comments were made	6.42
4. Strategies on how to avoid common errors and biases in the clinical reasoning process	The interviewees mentioned specifically also the lack of error culture at their institutions	6.41
5. Teaching methods for face-to-face courses (e.g., seminars, problem-based learning courses, lectures)	No comments were made	6.41
6. Assessment methods of clinical reasoning	Assessment methods are seen as important by the interviewees so that teachers can determine if their teaching was effective	6.37
7. Technology-enhanced methods (such as virtual patients, e-learning, etc.)	The interviewees see this as useful but also somewhat problematic because of a lack of needed infrastructure for such methods at some institutions	6.13
8. Blended learning / Flipped (inverted) classroom methodology	No comments were made	6.02
9. Theory on clinical reasoning	The interviewees find it important, teachers should know the theory, but it should also be related to practice	5.82
10. Literature on clinical reasoning	The interviewees also see literature as less important for this purpose and should be integrated to a minimal extent	5.73

^a^Note: Scale: 7 = Very important; 6 = Important; 5 = Somewhat important; 4 = Neutral; 3 = Rather unimportant; 2 = Unimportant; 1 = Very unimportant

Finally, the interviewees were asked about the ideal length of a clinical reasoning TTT-course. The answers varied greatly from 2–3 hours to a two-year educational program, with a tendency toward 1–2 days. Several interviewees commented that the time teachers are able to spend on a TTT-course is limited. This should be considered in the planning of such a course to make participation feasible for teachers.

## Discussion

In this study, we investigated the current status of and suggestions for teaching and assessment of clinical reasoning (CR) in a longitudinal curriculum as well as suggestions for a train-the-trainer (TTT-) course for CR. Teachers and students were invited to participate in online-surveys as well as semi-structured interviews to derive answers to our research questions. Regarding the contents of a CR curriculum for students, the results of the surveys and interviews were comparable and favoured content such as gathering, interpreting, and synthesizing patient information, generating differential diagnoses, and developing a diagnostic and a treatment plan. In the interviews, high importance was additionally placed on collaborative and interprofessional aspects of CR. Case-based learning and simulations were seen as the most useful methods for teaching CR, and clinical and oral examinations were favoured for the assessment of CR. The preferred format for a TTT-course was blended learning. In terms of course content, teaching and assessment methods for CR were emphasized. In addition to research from the North American region [11], this study provides results from predominantly European countries that support the existing findings.

### Content of a clinical reasoning curriculum

Our results revealed that there are still aspects of clinical reasoning (CR), such as “Errors in the clinical reasoning process and strategies to avoid them” or “Interprofessional aspects of CR” that are rarely taught despite their high importance, corroborating the findings of Kononowicz et al. [10]. According to the interviewees, students should have basic knowledge of CR before they are taught about errors in the CR process and strategies to avoid them. The lack of teaching of errors in CR may also stem from a lack of institutional culture regarding how to manage failures in a constructive way (e.g. [16, 25]), making it difficult to explicitly address errors and strategies to avoid them. Although highly relevant in the everyday practice of healthcare professions and underpinned by CR theoretical frameworks (e.g., distributed cognition [26]), interprofessional and collaborative aspects of CR are currently rarely considered in the teaching of CR. The interviews suggested that hierarchical distance and cultural barriers may contribute to this finding. Sudacka et al. [16] also reported cultural barriers as one reason for a lack of CR teaching. Generally, the interviewees seemed to place greater importance on interprofessional and collaborative aspects than did the survey-participants This may have been due to differences in the professions represented in the two modalities (e.g., a greater percentage of nurses among the interview participants, who tend to define CR more broadly than physicians [5]).

“Self-reflection on clinical reasoning performance and strategies for future improvement”, “Developing a problem formulation/hypothesis” and “Aspects of patient-participation in CR” were rated as important but are currently rarely taught, a finding not previously reported. The aspect “Self-reflection on clinical reasoning performance and strategies for future improvement”, received high importance ratings, but only 25% of the survey-participants answered that it is currently taught to a great extent. The interviewees agreed that self-reflection is important and added that ideally, it should be guided by specific questions. Ogdie et al. [27] found that reflective writing exercises helped students identify errors in their reasoning and biases that contributed to these errors.

“Gathering, interpreting, and synthesizing patient information” and “Developing a problem formulation/hypothesis” were rated significantly more important by teachers than by students. It appears that students may be less aware yet of the importance of gathering, interpreting, and synthesizing patient information in the clinical reasoning process. There was some indication in the interviews that the students may not have had enough experience yet with “Developing a problem formulation/hypothesis” or associate this aspect with research, possibly contributing to the observed difference.

Overall, our results on the contents of a CR curriculum suggest that all content is important and should be included in a CR curriculum, starting with basic theoretical knowledge and data gathering to more advanced aspects such as errors in CR and collaboration. Two other recent surveys conducted in the United States among pre-clerkship clinical skills course directors [12] and members of clerkship organizations [13] came to similar conclusions regarding the inclusion of clinical reasoning content at various stages of medical curricula. How to fit the content into already dense study programs, however, can still be a challenge [16].

### Clinical reasoning teaching methods

In addition to case-based learning and clinical teaching, human simulated patients and Team-based learning also received high importance ratings for teaching clinical reasoning (CR), a finding not previously reported. Lectures, on the other hand, are seen as the least important to teach CR (see also Kononowicz et al. [10]), as they mainly deliver factual knowledge according to the interviewees. High-fidelity simulations (mannequins) and Virtual Patients (VPs) are rarely used to teach CR at the moment and are rated less important compared to other teaching formats. Some interviewees see high-fidelity simulations as more useful for teaching practical skills. The lower importance rating of VPs was surprising given that this format is case-based, provides a safe environment for learning, and is described in the literature as a well-suited tool for teaching CR [28, 29]. Considering that VPs seemed to be used less often at the institutions involved in this study, the lack of experience with this format may have led to this result.

Teachers rated Team-based learning as significantly more important for teaching clinical reasoning than students. In the interviews, many students seemed not to be familiar with Team-based learning, possibly explaining the lower ratings the students gave this format in the survey.

Taken together, our results suggest that there is not one best format for teaching all aspects of clinical reasoning but rather that the use of all teaching formats is justified depending on the specific content to be taught and goals to be achieved. However, there was agreement that a safe learning environment where no patients can be harmed is preferred for teaching clinical reasoning, and that discussions should be possible.

There was wide agreement that clinical reasoning (CR) teaching should start in the first year of study in the curriculum. However, a few participants of this study argued that students first need to develop some general knowledge before CR is taught. Rencic et al. [11] reported that according to internal medicine clerkship directors, CR should be taught throughout all years of medical school, with a particular focus during the clinical teaching years. A similar remark was made by participants in a survey among pre-clerkship clinical skills course directors by Gupta et al. [12] where the current structure of some curricula (e.g. late introduction of the pathophysiology) was regarded as a barrier to introducing CR from the first year of study on [12].

### Assessment of clinical reasoning

Our results show that the most important format for assessing clinical reasoning (CR) that is also currently used to the greatest extent are clinical examinations (e.g. OSCE), consistent with Kononowicz et al. [10]. The interviewees emphasized that CR should ideally be assessed in a conversation or discussion where the learners can explain their reasoning. Given this argument, all assessment formats enabling a conversation are suitable for assessing CR. This is reflected in our survey results, where assessment formats that allow for a discussion with the learner received the most favourable importance ratings, including oral examinations. In agreement with Kononowicz et al. [10], we also found that written tests are currently used most often to assess CR but are rated as least important and suitable only for the assessment of some aspects of CR. Daniel et al. [3] argued that written exams such as MCQs, where correct answers have to be selected from a list of choices, are not the best representation of real practical CR ability. Thus, there still seems to be potential for improvement in the way CR is assessed.

Teachers rated clinical examinations and workplace-based assessments significantly higher than students. Based on the interviews, the students seemed to associate clinical examinations such as OSCEs more with a focus on practical skills than CR, potentially explaining their lower ratings of this format.

### What a clinical reasoning train-the-trainer course should look like

Our results show a clear need for a clinical reasoning (CR) train-the-trainer course (see also Singh et al. [15]), which currently does not exist at most institutions represented in this study, corroborating findings by Kononowicz et al. [10]. A lack of adequately trained teachers is a common barrier to the introduction of CR content into curricula [12, 16]. According to our results such a course should follow a blended learning/flipped classroom approach or consist of a series of face-to-face meetings. A blended-learning course would combine the benefits of both self-directed learning and the possibility for trainers to discuss with and learn from their peers, which could also increase their motivation to participate in such a course. An e-learning only course or a one-time face-to-face meeting were considered insufficient. The contents “Clinical reasoning strategies” and “Common errors in the clinical reasoning process” were given greater importance for the trainer-curriculum than for the students-curriculum, possibly reflecting higher expectations of trainers as “CR experts” compared with students. There was some agreement in the interviews that ideally, the course should not be too time-consuming, with participants tending towards an overall duration of 1–2 days, considering that most teachers usually have many duties and may not be able or willing to attend the course if it were too long. Lack of time was also identified as a barrier to attending teacher training [12, 13, 16].

## Strengths and limitations

The strengths of this study include its international and interprofessional participants. Furthermore, we explicitly included teachers and students as target groups in the same study, which enables a comparison of different perspectives. Members of the target groups not only participated in a survey but were also interviewed to gain in-depth knowledge. A distinct strength of this study is its mixed-methods design. The two data collection methods employed in parallel provided convergent results, with responses from the web survey indicating global needs and semi-structured interviews contributing to a deeper understanding of the stakeholder groups’ nuanced expectations and perspectives on CR education.

This study is limited in that most answers came from physicians, making the results potentially less generalizable to other professions. Furthermore, there were participants from a great variety of countries, with some countries overrepresented. Because of the way the survey-invitations were distributed, the exact number of recipients is unknown, making it impossible to compute an exact response rate. Also, the response rate of the survey was rather low for individuals who opened the survey. Because the survey was anonymous, it cannot completely be ruled out that some individuals participated in both interviews and survey. Finally, there could have been some language issues in the interview analysis, as the data were translated to English at the local partner institutions before they were submitted for further analysis.

## Conclusion

Our study provides evidence of an existing need for explicit clinical reasoning (CR) longitudinal teaching and dedicated CR teacher training. More specifically, there are aspects of CR that are rarely taught that our participants believe should be given priority, such as self-reflection on clinical reasoning performance and strategies for future improvement and aspects of patient participation in CR that have not been previously reported. Case-based learning and clinical teaching methods were again identified as the most important formats for teaching CR, while lectures were considered relevant only for certain aspects of CR. To assess CR, students should have to explain their reasoning, and assessment formats should be chosen accordingly. There was also still a clear need for a CR train-the-trainer course. In addition to existing research, our results show that such a course should ideally have a blended-learning format and should not be too time-consuming. The most important contents of the train-the-trainer course were confirmed to be teaching methods, CR strategies, and strategies to avoid errors in the CR process. Examples exist for what a longitudinal CR curriculum for students and a corresponding train-the-trainer course could look like and how these components could be integrated into existing curricula (e.g. DID-ACT curriculum [20], https://did-act.eu/integration-guide/ or the described curriculum of Singh et al. [15]). Further research should focus on whether and to what extent the intended outcomes of such a curriculum are actually reached, including the potential impact on patient care.

## Data Availability

All materials described in this manuscript generated during the current study are available from the corresponding author on reasonable request without breaching participant confidentiality.

## Appendix

**Table 8:** Questions in the surveys and interviews

Question	Surveys	Interviews
*Demographics*		
In which country do you work/study?	x	x
What educational programme do you relate mostly to?	x	x
*Student’s Curriculum*		
Please rate the importance of inclusion of each of the following aspects in the envisioned longitudinal curriculum on clinical reasoning*	x	x
1. Gathering, interpreting, and synthesizing patient information		
2. Generating differential diagnoses including defining and discriminating features		
3. Developing a diagnostic plan		
4. Developing a treatment/management plan		
**5. Developing a problem formulation/hypothesis**		
6. Errors in the clinical reasoning process and strategies to avoid them		
**7. Self-reflection on clinical reasoning performance and strategies for future improvement**		
8. Theories of clinical reasoning (e.g. knowledge encapsulation, illness scripts, narrative reasoning)		
9. Strategies to learn clinical reasoning (e.g. heuristics, rule out worst case scenario)		
**10. Collaborative aspects of clinical reasoning**		
11. Interprofessional aspects of clinical reasoning		
**12. Aspects of patient participation in clinical reasoning (e.g. shared decision making)**		
Please rate the importance of inclusion of each of the following formats in the envisioned longitudinal curriculum on clinical reasoning^*^	x	x
1. Lectures		
2. Problem Based Learning (PBL)		
**3. Case-based Learning**		
**4. Team-based Learning**		
5. Virtual Patients (interactive online cases)		
**6. High-fidelity simulation (mannequins)**		
**7. Human simulated patients**		
From which study year on should clinical reasoning be taught in the envisioned longitudinal curriculum on clinical reasoning?	x	x
1. Year 1		
2. Year 2		
3. Year 3		
4. Year 4		
5. Year 5		
6. Year 6		
*Assessment format*		
Which of these assessment formats should be implemented in the envisioned longitudinal curriculum on clinical reasoning?^a^	x	x
1. Written test (e.g. multiple choice questions, key feature approach, script concordance tests)		
**2. Oral examination**		
**3. Assessment using virtual patients**		
4. Clinical examinations (e.g. OSCE or other practical examinations)		
5. Workplace-based assessments (e.g. MiniCEX, summative approach)		
*Present clinical reasoning curriculum*		
In your curriculum (i.e. overall programme, not a particular course or clerkship you might be overseeing), which of the following aspects are taught and assessed?^b^	x	x
1. Gathering, interpreting, and synthesizing patient information		
2. Generating differential diagnoses including defining and discriminating features		
3. Developing a diagnostic plan		
4. Developing a treatment/management plan		
**5. Developing a problem formulation/hypothesis**		
6. Errors in the clinical reasoning process and strategies to avoid them		
**7. Self-reflection on clinical reasoning performance and strategies for future improvement**		
8. Theories of clinical reasoning (e.g. knowledge encapsulation, illness scripts, narrative reasoning)		
9. Strategies to learn clinical reasoning (e.g. heuristics, rule out worst case scenario)		
**10. Collaborative aspects of clinical reasoning**		
11. Interprofessional aspects of clinical reasoning		
**12. Aspects of patient participation in clinical reasoning (e.g. shared decision making)**		
How is clinical reasoning TAUGHT in your curriculum (i.e. overall programme, not a particular course or clerkship you might be overseeing) in sessions with a main focus on clinical reasoning?^b^	x	x
1. Lectures		
2. Problem Based Learning (PBL)		
**3. Case-based Learning**		
**4. Team-based Learning**		
5. Virtual Patients (interactive online cases)		
**6. High-fidelity simulation (mannequins)**		
**7. Human simulated patients**		
From which study year on is clinical reasoning taught at your institution?	x	x
1. Year 1		
2. Year 2		
3. Year 3		
4. Year 4		
5. Year 5		
6. Year 6		
How is clinical reasoning ASSESSED in your curriculum?^b^	x	x
1. Written test (e.g. multiple choice questions, key feature approach, script concordance tests)		
**2. Oral examination**		
**3. Assessment using virtual patients**		
4. Clinical examinations (e.g. OSCE or other practical examinations)		
5. Workplace-based assessments (e.g. MiniCEX, summative approach)		
Do you have a train-the-trainer course on clinical reasoning at your institution?	x	x
1. Yes		
2. No		
3. I don’t know		
If yes, please describe:		
*Train-the-trainer curriculum*		
Do you think the DID-ACT train-the-trainer course is necessary for healthcare educators at your institution?	x	x
1. Yes		
2. No		
3. I don’t know		
What should the DID-ACT train-the-trainer course on clinical reasoning cover?^a^	x	x
1. Literature on clinical reasoning		
2. Theory on clinical reasoning		
3. Clinical reasoning strategies		
4. Common errors in the clinical reasoning process		
5. Strategies on how to avoid common errors and biases in clinical reasoning process		
6. Teaching methods on the wards and/or clinic		
7. Teaching methods for face-to-face courses (e.g. seminars, problem-based learning courses, lectures)		
8. Technology-enhanced methods (such as virtual patients, e-learning		
**9. Blended learning / Flipped (inverted) classroom methodology**		
10. Assessment methods of clinical reasoning		
In your opinion, what is the best format for the DID-ACT train the trainer course?	x	x
1. One time face-to-face meeting		
2. Series of face-to-face meetings		
3. E-learning course		
4. Blended learning/flipped classroom approach (combination of e-learning and face-to-face meetings)		
Why do you suggest the format above for the train-the-trainer course? Please explain	x	x
How much time (e.g. days/hours) would you be willing to spend on a train-the-trainer course?		x
Do you have further suggestions for the DID-ACT train-the-trainer course?	x	x

^a^Scale: 1 = Very important; 2 = Important; 3 = Somewhat important; 4 = Neutral; 5 = Rather unimportant; 6 = Unimportant; 7 = Very unimportant; 8 = I don’t know

^b^Scale: 1 = To a great extent; 2 = To some extent; 3 = A little; 4 = Not at all; 5 = I don't know; Bold are aspects that were added/adapted in comparison to Kononowicz et al.[10]

**Table 9:** Survey: Contents that are currently being taught

Question: In your curriculum, which of the following aspects are taught?	To a great extent (%)	To some extent (%)	A little (%)	Not at all (%)
Gathering, interpreting, and synthesizing patient information	59.1	34.1	6.8	–
Developing a treatment/management plan	54.5	36.4	9.1	–
Developing a diagnostic plan	54.5	34.1	9.1	2.3
Generating differential diagnoses including defining and discriminating features	53.3	37.8	8.9	–
Developing a problem formulation/hypothesis	33.3	44.4	20.0	2.2
Aspects of patient participation in clinical reasoning (e.g. shared decision making)	31.8	40.9	20.5	6.8
Self-reflection on clinical reasoning performance and strategies for future improvement	25.0	30.0	27.5	17.5
Strategies to learn clinical reasoning (e.g. heuristics, rule out worst case scenario)	19.5	31.7	34.1	14.6
Errors in the clinical reasoning process and strategies to avoid them	16.7	40.5	23.8	19.0
Interprofessional aspects of clinical reasoning	14.0	37.2	32.6	16.3
Collaborative aspects of clinical reasoning	14.0	34.9	37.2	14.0
Theories of clinical reasoning (e.g. knowledge encapsulation, illness scripts, narrative reasoning)	2.3	39.8	37.2	20.9

7 = Very important; 6 = Important; 5 = Somewhat important; 4 = Neutral; 3 = Rather unimportant; 2 = Unimportant; 1 = Very unimportant

**Table 10:** Survey: Current teaching formats of clinical reasoning

Question: How is clinical reasoning taught in your curriculum in sessions with a main focus on clinical reasoning?	To a great extent (%)	To some extent (%)	A little (%)	Not at all (%)
Case-based Learning	44.4	37.8	17.8	–
Problem Based Learning (PBL)	26.2	40.5	7.1	26.2
Human simulated patients	25.6	37.2	20.9	16.3
Team-based Learning	16.7	50.0	23.8	9.5
Lectures	15.9	29.5	45.5	9.1
Virtual Patients (interactive online cases)	7.3	43.9	26.8	22.0
High-fidelity simulation (mannequins)	7.3	36.6	36.6	19.5

7 = Very important; 6 = Important; 5 = Somewhat important; 4 = Neutral; 3 = Rather unimportant; 2 = Unimportant; 1 = Very unimportant

**Table 11:** Survey: Current assessment formats of clinical reasoning

Question: How is clinical reasoning assessed in your curriculum in sessions with a main focus on clinical reasoning?	Current: To a great extent (%)	To some extent (%)	A little (%)	Not at all (%)
Clinical examinations (e.g. OSCE or other practical examinations)	37.5	47.5	10.0	5.0
Workplace-based assessments (e.g. Mini-CEX, summative approach)	17.5	35.0	27.5	20.0
Oral examination	20.0	37.5	15.0	27.5
Assessment using virtual patients	15.4	23.1	28.2	33.3
Written test (e.g. multiple choice questions, key feature approach, script concordance tests)	47.5	32.5	12.5	7.5

7 = Very important; 6 = Important; 5 = Somewhat important; 4 = Neutral; 3 = Rather unimportant; 2 = Unimportant; 1 = Very unimportant

**Table 12:** Interviews: Current clinical reasoning contents and their importance for a future longitudinal curriculum

Current content areas	Summary of comments by teachers on future use	Summary of comments by students on future use
Gathering, interpreting, and synthesizing patient information - Dealing with information sources	Is seen as an important aspect. It is often mentioned in relation to history taking and seen as the foundation and a key element in the reasoning process	Is seen as the important basis of the CR process, which influences all the following steps
Generating differential diagnoses including defining and discriminating features	Is seen as an important ability for the students to develop	Important aspect they associate with the handling of data
Developing a treatment/management plan	Important part of the process that can be difficult for the students. Management after hospital discharge should also be covered. Furthermore, this aspect is seen as field-specific	Important, e.g. forward planning
Developing a diagnostic plan	This aspect is seen as rather important	A clear plan what has to be done, a structured procedure, is seen as important
Self-reflection on clinical reasoning performance and strategies for future improvement - Quality/evaluation of clinical reasoning	Self-reflection on clinical reasoning performance and strategies for future improvement are important to consider	Important, also for the learning process. The self-reflection should be guided, for example with specific questions
Errors in the clinical reasoning process and strategies to avoid them	Errors and how to avoid them is an important and central aspect that, however, should be taught after the students already have gathered basic knowledge on clinical reasoning	Very important and relevant aspect. The role of communication in errors is especially emphasized
Developing a problem formulation/hypothesis	Important part of the process	The students’ opinions are rather divided regarding this aspect. One person associates it more with research
Interprofessional aspects of clinical reasoning - Interprofessional communication	Very important and multifaceted aspect. Communication, clarification of roles, knowing when it is necessary to consult with other professions, professional identity, and the chance to learn from each other were mentioned. Could be taught with interprofessional cases. But not everything should be taught to all professions at once. Further, there still exists a hierarchical distance between professions. Cultural change is needed which may be difficult to bring about	Important aspect that should be more emphasized
Aspects of patient participation in clinical reasoning (e.g. shared decision making)	Generally seen as important. Some interview participants question whether the patient can/should be part of the clinical reasoning process. One person actively advises against teaching this aspect as a part of CR. Some other participants mention that shared decision making is not part of CR	Important aspect, the patient should be included according to the students
Collaborative aspects of clinical reasoning	Collaboration is seen as an important aspect. Especially communication and shared decision-making are emphasized	Important, especially regarding shared decision-making
Strategies to learn clinical reasoning (e.g. heuristics, rule out worst case scenario,…)	The teachers found this aspect somewhat important. Only heuristics have been mentioned twice in relation with this aspect	The students’ thoughts on this aspect differ greatly. No facet has been mentioned more than once
Theories of clinical reasoning (e.g. knowledge encapsulation, illness scripts, narrative reasoning …) - Systematic approach to clinical reasoning - Metacognitive decision making - Situational thinking	Is also mostly seen as a relevant aspect. An understanding of what clinical reasoning is should be established, but with practice-orientation	Do not put too much emphasis on theories

**Table 13:** Interviews: Importance of current clinical reasoning teaching formats for a future longitudinal clinical reasoning curriculum

Current formats	Summary of comments by teachers on future use	Summary of comments by students on future use
Clinical teaching - Bedside-teaching - Clinical work - Internships	Teaching in real clinical settings is seen as very important. This can be a very insightful experience for learners but needs to be well supervised and the teachers should be good examples. It could also be helpful to learn about the perspectives of other professions. The CR process should ideally be discussed with the supervisor(s)	The students did not mention clinical teaching formats
Case-based learning (CBL)	Very important format for the teaching of clinical reasoning. Multiple cases of varying complexity, where students have to think about the case more, should be used over time	Suitable format for teaching of clinical reasoning. Could also be used for interprofessional learning
Human simulated patients	Generally seen as an important format	Important/ useful format, but the SPs must be well trained. Some students also mentioned that this format is less useful because they know that the patients are not real
Team-based learning	Important format that allows for discussions and could also be used for teaching of interprofessional aspects of clinical reasoning	Some students did not know the format. However, most of them can imagine it to be suitable for the teaching of clinical reasoning
Problem-based learning (PBL)	Important format for the teaching of CR as it allows for a guided, safe environment for learning CR	Very suitable format for teaching CR, but it should be more than knowledge transfer / theory
High-fidelity simulation (mannequins)	The opinions are more divided regarding this format. Some see it as useful for advanced students, but also high preparatory effort is mentioned	Useful, but also a few critical aspects are mentioned like less process-orientation, more for practical skills
Virtual Patients (interactive online cases)	Future-oriented/important in the future, also more complex scenarios can be displayed. Could be used to prepare students for clinical work	Very useful format to try things out without harm
Lectures / Seminars	Lectures are important to establish a knowledge base and to show relations. Lectures could e.g. be enriched by video examples	Limited use, only for basic, factual knowledge
Blended learning	This could be for example combinations of lectures and Virtual Patients or of TBL and Virtual Patients	The students did not mention blended learning

**Table 14:** Interviews: Importance of current clinical reasoning assessment formats for a future longitudinal clinical reasoning curriculum

Current formats	Summary of comments by teachers on future use	Summary of comments by students on future use
Clinical examinations (e.g. Objective Structured Clinical Examination or other practical examinations) - During rounds	Suitable format. There should be a conversation or discussion so that the students have to explain their reasoning. However, there is also the question of suitable, objective evaluation criteria / dependence on the examiners' perception	More suitable for testing clinical skills, only some students find it useful for the assessment of CR
Workplace-based assessments (WBA) - Formative assessment - MiniCEX - Practical assessment - Patient care	Also in the context of WBAs, students can be well questioned about their CR, but according to some it should rather be used towards the end of the formal training of the students	Very suitable for the assessment of clinical reasoning because it is the closest to reality
Oral examinations	There should be a conversation or discussion in assessing CR. The students should explain their reasoning. Examiners should be trained	The students’ opinions were rather divided regarding oral exams with some questioning their objectivity and some fear that only factual knowledge would be tested. The examiners should be trained and ideally, feedback should be provided
Assessment using Virtual Patients (VPs)	VPs allow for objectivity and standardization, however, the teachers differ in their opinions whether they would be useful for assessment	A useful format, no patients can be harmed
Written tests - Multiple Choice-single best answer questions - Written case reflection - Progress Test - Script concordance test	Suitable format for some aspects of CR. Less preparatory effort needed than for other formats and also less for evaluating the results	Not the most useful format. CR is difficult to assess with Multiple Choice questions for example
